# The formation of egg granulomas in the spleens of mice with late *Schistosoma japonicum* infection alters splenic morphology

**DOI:** 10.1186/s13071-015-0988-x

**Published:** 2015-07-16

**Authors:** Yanjuan Wang, Jing Zhang, Jianhai Yin, Yujuan Shen, Ying Wang, Yuxin Xu, Jianping Cao

**Affiliations:** National Institute of Parasitic Diseases, Chinese Center for Disease Control and Prevention; Laboratory of Parasite and Vector Biology, MOH, Shanghai, China; WHO Collaborating Center for Malaria, Schistosomiasis and Filariasis, Shanghai, 200025 PR China

**Keywords:** *Schistosoma japonicum*, Spleen, Egg granulomas, Lymphoid follicles

## Abstract

**Background:**

Splenomegaly is a characteristic symptom of schistosome infection. Unlike the well known hepatic pathology of schistosomiasis, splenomegaly has received little scientific research and is generally considered to be a non-specific congestion caused by increased blood pressure within the venous sinuses. Moreover, to date, few studies have reported the deposition of schistosome eggs in the spleen. In a previous study, however, we observed that prolonged *S. japonicum* infections destroyed the structure of the lymphoid follicles in the spleen of mice at 8 weeks post-infection and found that eggs were frequently deposited in the spleen. These prior observations suggested a relationship between granulomas and splenic morphology which we investigate further in this study.

**Methods:**

C57BL/6 mice were infected percutaneously with twenty cercariae of *S. japonicum* and sacrificed at different times post-infection. The number of eggs present in the homogenates of spleens and livers was quantified by light microscopy. Splenic pathology was observed by immunohistochemistry staining of paraffin-embedded sections. At 18 weeks post-infection the infected mice were divided into two groups (granulomatous spleens and non-granulomatous spleens). Serum antibodies and cytokines in the antigen- or mitogen-stimulated lymphocyte cultures were then determined by ELISA.

**Results:**

We found that eggs deposition in the spleens of infected mice occurred frequently but only occasionally led to granulomas formation. The lymphoid follicles within the granulomatous spleens maintained their structural integrity until 20 weeks post-infection, unlike the lymphoid follicles in spleens without egg granulomas. Mice with granulomatous spleens accompanied by lymphoid follicles exhibited a germinal center (GC)-like structure and had enhanced humoral immune responses. Splenocytes from granulomatous spleens also showed significantly elevated levels of Th2 cytokines during late infection stages.

**Conclusions:**

Our results highlight that lymphoid follicles, which are not completely destroyed or are re-established in the spleen, can change the local immune environment and lead to changes in the splenic morphology of mice with chronic schistosomiasis.

## Background

It is well known that the eggs of schistosomes can be deposited in the liver and intestine of their vertebrate host where they induce inflammatory granulomas, which are the most characteristic pathological feature of schistosomiasis. However, schistosome eggs can sometimes be deposited in places besides organs associated with the hepatic portal vein where they can cause serious ectopic lesions (e.g. ectopic schistosomiasis *mansoni* in the dermis [1, 2], the nervous system [3, 4], or the reproductive system [5–7]. To date, little is known about the deposition of eggs in the spleen. Some previous studies proposed that schistosome eggs rarely deposit in spleen because of the potent phagocytizing ability of the mononuclear phagocyte system in the splenic sinus [8]. Hence, even if eggs reached the spleen, it was thought that the splenic cells still had the ability to inhibit the formation of egg-induced granulomas [9]. In a previous study [10] we showed that *S. japonicum* infection destroyed the structure of the lymphoid follicles in the spleen of mice 8 weeks post-infection. Our subsequent investigations revealed that eggs of *S. japonicum* can deposit in the spleen, though not all will initiate the formation of granulomas. In this study, we showed that as the infection progressed the architecture of lymphoid follicles seemed partially destroyed or had re-established in the spleen with granulomas. We also further explore the function of these accompanying lymphoid follicles to understand the process by which egg granulomas changed splenic morphology in late *S. japonicum* infection in mice.

## Methods

### Ethical statement

Animal care and all animal procedures were carried out according to the Guidelines for the Care and Use of Laboratory Animals produced by the Shanghai Veterinary Research Institute. The study was approved by the Ethics Committee of the National Institute of Parasitic Diseases, Chinese Center for Disease Control and Prevention.

### Animals and parasites

Male C57BL/6 mice (6–8 weeks old) were obtained from the Shanghai Laboratory Animal Center, Shanghai, China. *Oncomelania hupensis* snails harboring *S. japonicum* cercariae were purchased from the Jiangsu Institute of Parasitic Diseases (Wuxi, China). Mice were infected percutaneously with 20 cercariae of *S. japonicum* and sacrificed at different times following infection (6–14 mice at each time point). To observe the changes caused by splenic granulomas, at 18 weeks post-infection the infected mice were grouped by the gross morphology of the spleen (whether macroscopic granulomas were evident or not in the spleen after bleeding and dissection). Fifteen mice were sacrificed at 18 weeks post-infection, and seven of them were found to have granulomas in the spleen, while the other eight mice did not.

### Spleen histopathology

Spleens were embedded in paraffin and stained with hematoxylin and eosin (H&E) prior to microscopic examination. In some experiments, spleen samples were weighed and ground into homogenates in phosphate-buffered saline solution (PBS). All released eggs were quantified by light microscopy. We used liver samples as controls, which were treated as above, but were digested in 4 % potassium hydroxide. For each mouse, we calculated the total egg count in the liver through examination of three digestions of the same volume.

### Immunohistochemistry

To evaluate the function of lymph follicles in the spleens of mice with late-stage *S. japonicum* infections, the GCs were detected by staining of the proliferation marker Ki67 (BD, Franklin Lakes, NJ, USA). Briefly, paraffin-embedded spleen sections were de-paraffinized and repaired. Endogenous peroxidase was quenched by incubating the slides for 30 min in PBS 0.3 % H2O2. The sections were first incubated with anti-mouse ki67, and then incubated with biotinylated anti-Ig in HRP Detection Kit (BD, Franklin Lakes, NJ, USA). All antibody incubations were conducted for one hour at 37 °C in a humidified box after dilution. Horseradish peroxidase (HRP) activity was developed using diaminobenzidine (DAB). Nuclei were counter-stained with hematoxylin.

### Detection of serum antibodies

Soluble egg antigen (SEA) was prepared as described previously [11]. The protein concentration was determined using the BCA Protein Assay kit (Bio-Rad, Richmond, CA, USA). Serum levels of SEA specific IgG or IgM were determined by ELISA. Briefly, 96-well plates were coated overnight at 37 °C with 5 μg/μL SEA, after washing and blocking. We then added serum samples or serum dilution buffer. Serum IgG or IgM binding to the plates was measured with HRP-conjugated anti-mouse IgG or IgM, respectively. Color development was determined by measuring the absorbance at 450 nm. All antibodies were purchased from Santa Cruz Biotechnology.

### Cytokine assay

Splenic lymphocytes were obtained from infected mice using lymphocyte seperation medium (Dakewe, Shenzhen, China). Cell viability was always >95 %, as determined by the trypan blue exclusion method and adjusted to 10^6^ cells/mL. Cells were cultured for 48 h in an RPMI 1640 medium containing 10 % FBS and either 15 μg/mL SEA or 10 μg/mL concanavalin A (ConA). Supernatants were harvested and used to analyze cytokines levels of IFN-γ, IL-2, IL-4, IL-5, IL-10 and IL-17. All of the cytokines were measured using commercially available kits (IFN-γ, IL-2, IL-4, IL-5, IL-17: Biolegend, San Diego, CA, USA; IL-10: R&D System, Minneapolis, MN, USA)

### Statistical analyses

Statistical differences between groups were evaluated by the ANOVA and Kruskal-Wallis test using SPSS software (version 20.0; SPSS, Chicago, IL, USA) Statistical significance was considered at *P* < 0.05.

## Results

### Rate of egg deposition in the spleen

**Fig. 1:**
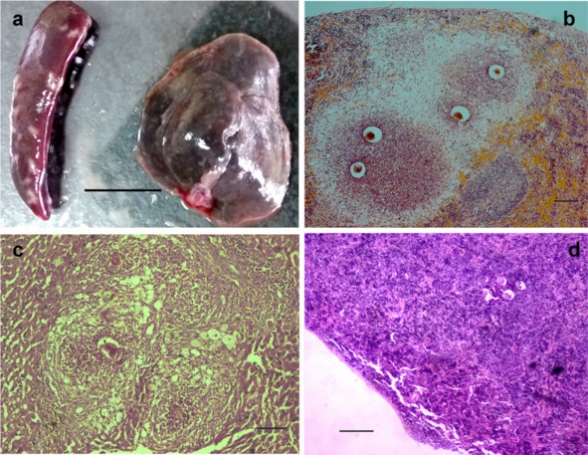
*S. japonicum* eggs deposited in the spleen. **a** Spleen and liver from mice at 8 weeks post-infection. **b** Paraffin section of a spleen with granulomas prepared from an infected mouse at 8 weeks post-infection and stained with H&E. **c** Paraffin section of liver from an infected mouse at 8 weeks post-infection and stained with H&E. **d** Paraffin section of spleen without granulomas prepared from an infected mouse at 18 weeks post-infection and stained with H&E. Between six and eight mice were observed at each time point. Scale bar: **a** 10 mm; **b** and **c** 100 μm; **d** 200μm

During the examination of spleen histopathology, egg-induced granuloma nodes were occasionally seen on the surface of the spleen. We assessed the spleen homogenates from 5 weeks post-infection (which was the earliest observation of egg deposition in the liver). Surprisingly, we found that egg deposition in the spleen of C57BL/6 mice was a common phenomenon (Table 1). Egg deposition in the spleen began later than in the liver (at 7 weeks rather than 5 weeks post-infection), increased gradually as infection progressed, but was much lower than in the liver at the same time points (Table 1). The formation and development of the granulomas in spleen also lagged behind that in the liver. While liver granulomas showed reduced size and alleviated pathology in mice at 8 weeks post-infection (Fig. 1a, 1c), spleen granulomas still appeared to be in the acute phase with inflammatory cells surrounding the eggs (Fig. 1a, 1b). Eggs were also found in the spleen without inducing granuloma nodes, even in mice at 18 weeks post-infection (Fig. 1d).

**Table 1:** Rate of deposition of *S. japonicum* eggs in the spleens and livers of test mice

Weeks post infection	No. of mice	No. of mice with eggs (eggs, Epg^a^)	
		in liver	in spleen
5	8	8 (13,447 ± 1170 )	0 (0)
6	8	8 (23,833 ± 2123)	0 (0)
7	8	8 (28,528 ± 4383)	3 (269 ± 67)
8	8	8 (30,760 ± 4146)	6 (414 ± 189)
12	7	7 (30,085 ± 4213 )	6 (522 ± 231 )

^a^Epg: eggs per gram (mean ± SD)

### Lymphoid follicles reappear in spleens accompanied by the formation of granulomas

**Fig. 2:**
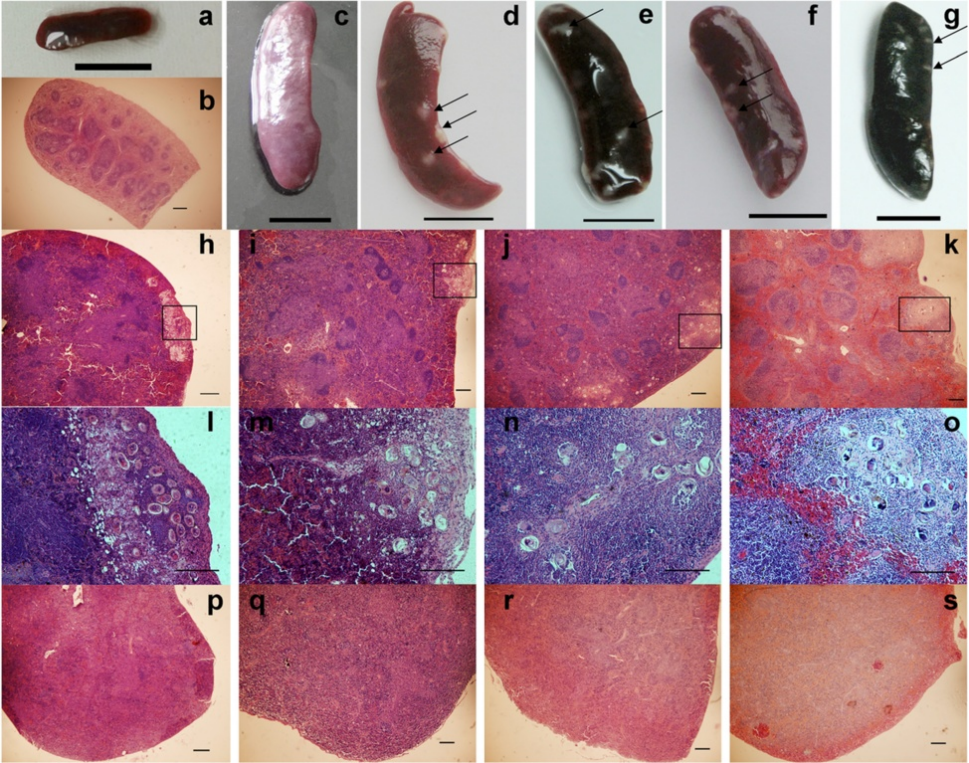
Lymphoid follicles reappear in spleens accompanied by the formation of granulomas in the later stage of infection. Spleen (**a**) and paraffin section of spleen (**b**) from naïve mice were used as controls. **c** Spleen without granulomas from infected mice 16 weeks post-infection. Spleen with granulomas from infected mice at **d** 8 weeks, **e** 12 weeks, **f** 16 weeks and **g** 20 weeks post-infection. Paraffin sections of spleen with granulomas prepared from infected mice at **h** 8 weeks, **i** 12 weeks, **j** 16 weeks and **k** 20 weeks post-infection, all stained with H&E. The area marked by the rectangles in panels (**h**–**k**) can be seen at higher magnification in panels (**l**–**o**), respectively. Paraffin sections of spleen without granulomas prepared from mice at **p** 8 weeks **q** 12 weeks, **r** 16 weeks and **s** 20 weeks post-infection, all stained with H&E. Between six and eight mice were observed at each time point. Similar results were obtained in each group. Egg granulomas are indicated by black arrows. Scale bar: **a**, **c** – **g** 10 mm; **b**, **h**–**s** 200 μm

Splenomegaly was evident in all infected mice for the whole chronic infection period from 8 weeks post-infection, though the size of the granuloma nodes formed on the spleen decreased gradually as infection progressed (Fig. 2d–g). Granulomas could not be seen on all spleens from mice of the same group (Fig. 2c). The paraffin sections stained with H&E showed that the spleen granulomas also changed from an acute phase to a chonic phase between 8 and 16 weeks post-infection, similar to the changes seen in liver pathology (Fig. 2l–o). Interestingly, the structure of the lymphoid follicles on the spleens, which were originally destroyed, seemed to re-establish or be maintained during later stages of infection (Fig. 2h–k). However, no follicle structure was seen on the non-granulomatous spleens from the same groups of mice at the same time points after infection (Fig. 2p–s).

### Functional characterization of lymphoid follicles in the granulomatous spleen

**Fig. 3:**
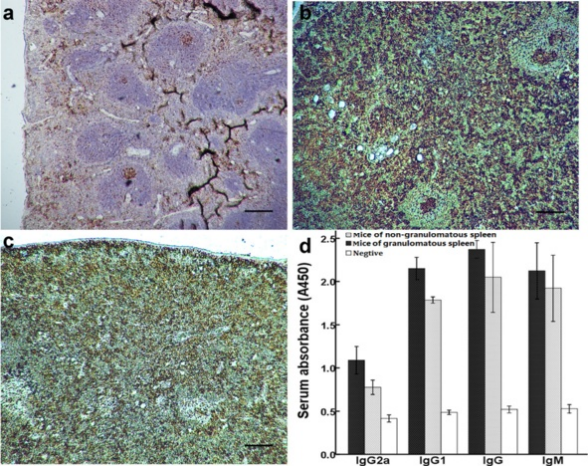
Functional characterization of lymphoid follicles in the granulomas spleen determined by immunohistochemistry on paraffin-embedded sections of spleen stained with antibody against Ki67. **a** Naïve (uninfected) mice used as control, **b** spleen with granulomas, **c** spleen without granulomas. **d** serum SEA specific IgG and IgM (1:50 dilutions) measured by ELISA. The groups were mice with granulomatous spleens (*n* = 7), non-granulomatous spleens (*n* = 8) and naïve mice (*n* = 8). Bars indicate means ± standard deviations. Scale = 200 μm

To verify if the lymphoid follicles influnced humoral immunity during late infection stages, we examined proliferating lymphocytes (via Ki-67 staining) in a group of mice at 18 weeks post-infection. Paraffin sections of the spleens showed that splenomegaly of schistosomiasis was of a lympho-proliferative type rather than a congestive one. The lymphoid follicles in the granulomatous spleen exhibited active centers (Fig. 3b) that appeared more compact than the germinal centers (GCs) in the spleens of naïve mice (Fig. 3a). No architecture of follicles or active centers was observed in the spleens without granulomas (Fig. 3c). GCs are known to be the site of the memory B-cells that are generated and associated with T-dependent antibody responses [12]. Accordingly, we also detected that the serum levels of SEA specific IgG or IgM, and the levels of IgG and its subtypes IgG1, IgG2a and IgM, were all higher in mice with granulomatous spleens compared to mice with non-granulomatous spleens (though this difference was not statistically different, Fig. 3d).

### Th2 cytokines are greater in lymphocytes from granulomatous spleens during late infection stages

**Fig. 4:**
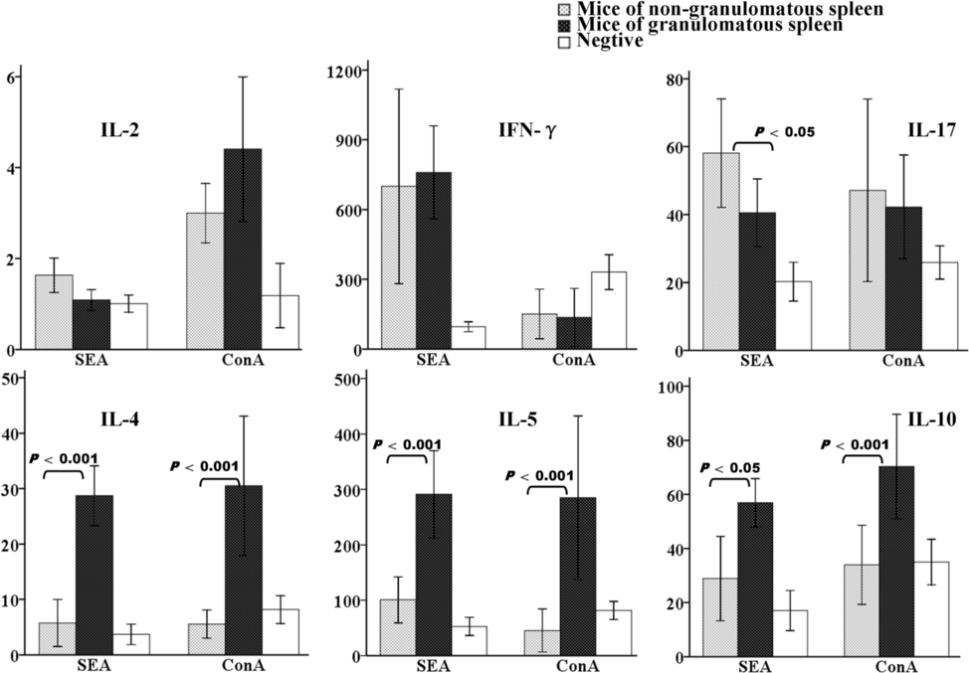
Cytokine responses in infected mice at 18 weeks post-infection stimulated by SEA or conA. Groups are mice with granulomatous spleens (*n* = 7), non-granulomatous spleens (*n* = 8) and naïve mice (*n* = 8). Bars indicate means ± standard deviations and statistically significant differences between the compared groups are shown as *p*-values

We found that levels of IL-4, IL-5 and IL-10 in the supernatants of splenic cells derived from granulomatous spleens were significantly higher than in cells derived from non-granulomatous spleens (Fig. 4). This was true both in the presence of SEA and ConA. In contrast, levels of IL-2 and IFN-γ were not significantly different between the groups (Fig. 4). Notably, levels of IL-17, a cytokine associated with hepatic or bladder pathology in schistosomiasis [13, 14], were higher in the supernatants secreted by splenocytes derived from non-granulomatous spleens (Fig. 4). In addition, we found that SEA and ConA differed in their ability to stimulate the production of type 1 cytokines (Fig. 4).

## Discussion

Granulomas lesions that form around eggs deposited in the liver is a common pathological change in various types of schistosomiasis and has been the focus of intense study [15–17]. Although the spleen is also one of the major organs affected during schistosomiasis, its pathology has long been overlooked. To date, little is known about rates of egg deposition in the spleen aside from a few cases reported as a result of heterotopic parasitism [8, 18]. In this study, we have shown that C57BL/6 mice infected with *S. japonicum*, commonly exhibit egg deposition accompanied by granulomas development in the spleen. Earlier studies had proposed that schistosome eggs deposited in different organs evoke different responses [19] and that the mononuclear phagocyte system in the splenic sinus has potent phagocytizing ability. This was suggested to explain why the presence of schistosome eggs and formation of granulomas in the spleen was seemingly less frequent. The result of this study, however, revealed that the deposition of schistosome eggs in the spleen is a common phenomenon as infection progresses.

Egg deposition constitutes the major pathogenesis of schistosomiasis, with egg-laying typically beginning 4 weeks after infection and reaching a peak at 8 weeks post-infection [17]. As the disease progresses from the acute to the chronic stage, granulomas decrease in both size and cellularity as a result of immune regulation. In this study, we found that eggs deposition and the formation of granulomas began later in the spleen than is typical for the hepatic organs (at 7 and 8 weeks post-infection, respectively). This observation may explain why this apparently common phenomenon has been relatively ignored to date. We assume this delay is simply associated with the different location of the vascular systems of the two organs. Interestingly, we observed that granulomas frequently formed in the cortex of the spleen but not in the medulla. Moreover, eggs deposited in the medulla did not induce granulomas even at 18 weeks post-infection.

Another unexpected finding was that lymphoid follicles were maintained within granulomatous spleens during later stages of infection. Lymphoid follicles in the spleen disappear during the chronic-phase of schistosomiasis via the reduction of splenic lymphocytes, through apoptosis [10] or their migration from the spleen to the hepatic egg granulomas [20]. Alternatively, the increase in monocytes concurrent with splenomegaly can also destroy the splenic architecture [21]. In this study, we observed that throughout the period of infection, mice experiencing the same infection conditions showed different spleen pathologies. Our results revealed that this heterogeneity was associated with the formation of splenic egg granulomas, suggesting that the granulomas play an essential role in changing the local immune environment in the spleen. However, we were not able to ascertain whether the lymphoid follicles re-established or had remained on the spleen during infection. Nevertheless, potent chemotactic activity in the granulomatous spleen should be involved in the aggregation of lymphocytes [22, 23]. Previous studies have shown that SEA, especially from *S. japonicum*, has prominent leucocyte chemotactic activity [24]. On the other hand, liver granulomas induce increased local chemokine expression, which is conversely correlated with altered chemotactic activity of splenic lymphocytes [21]. It has been proven that granulomas in the liver have the ability to recruit and eliminate activated splenic antigen-reactive lymphocytes, in order to protect host tissues from the pathological consequences of overproduction of inflammatory cytokines, explaining why granulomas are regarded to be an immune-regulatory organelle [25]. Granulomas in the spleen, however, may either recruit new lymphocytes to rebuild the lymphoid follicles or retain lymphocytes when competing for activated lymphocytes with the granulomas in liver. This closely resembles the ectopic secondary lymph follicles (SLF) that emerge near the filarial nodule [26] or tuberculous granulomas [27]. These are very effective to produce an SLF involving strong humoral responses at the site of infection, establishment and sustenance of this cellular organization comprise the infiltration of profound numbers of leukocytes and subsequent functional impairment of the local tissue, but whether splenic function was destroyed or strengthened in this study remains unclear.

Lymphoid follicles are important for the development and functional maturity of lymphocytes and are essential for mounting an efficient immune response [28]. Hence, ectopic SLF are usually found in autoimmune diseases [29, 30], and play a role in expanded inflammation and local defense. In this study, we found that the mice with granulomatous spleens, with accompaning lymphoid follicles, exhibited structures like GCs and had an enhanced humoral immune response. Indeed, cytokine levels suggested that the spleen granulomas were involved in creating an immune micro-environment that was characterized by a prominent Th2 response. Switching from Th1 to Th2 granuloma response is known to be a hallmark of chronic schistosomiasis. In addition, Th2 cells can alleviate inflammatory responses by secreting immune-regulatory cytokines when the host immune system fails to eliminate the pathogen [31]. Moreover, granuloma cells characterized by heightened production of IL-10 were found to suppress type 1 immune activation in mycobacterial granulomas, which has long been believed to be an active Th1 immune environment [32]. Of note, we observed significantly increased levels of IL-17 (a recognized pro-inflammatory cytokine) after SEA stimulation in mice with non-granulomatous spleens. Taken together, our results suggest that splenic granulomas accompanied by lymphoid follicles in mice with late-stage schistosomiasis may play a role in local defense rather than inflammation.

We were unable to determine which chemokines were involved in the re-establishment of follicles and whether they affected mice mortality, nor the underlying immune mechanisms of this process. Nevertheless, our results suggest that *S. japonicum* infection causes not only splenomegaly but also complicated changes in splenic morphology and the local immune environment. A better understanding of the role of spleen in this disease will assist in the clinical treatment of advanced hepatosplenic schistosomiasis.

## Conclusions

The spleen is the biggest peripheral immune organ and plays a vital role in the immune response to blood circulation. Although it is one of the major organs affected by schistosomiasis, its pathology has been long regarded as being linked to portal hypertension. In a previous study, we showed that *S. japonicum* infection destroyed the structure of the lymphoid follicles in the spleen of infected mice 8 weeks post-infection. In this study, we found that egg deposition in the spleen was a frequent occurrence in mice infected with *S. japonicum*, but occurred much later than is typical for hepatic organs. We also found that mice treated with the same infection conditions showed different spleen pathologies, and that this heterogeneity was associated with the formation of splenic egg granulomas. These results suggest that granulomas play an essential role in changing the local immune environment within the spleen.
